# Wolf Creek XVIII Part 3: Innovations in Defibrillation Science^☆^

**DOI:** 10.1016/j.resplu.2026.101229

**Published:** 2026-01-18

**Authors:** Rudolph W. Koster, Peter J. Kudenchuk, Sheldon Cheskes, Giuseppe Ristagno, Gregory P. Walcott

**Affiliations:** a Amsterdam UMC location University of Amsterdam, Heart Center, Department of Clinical and Experimental Cardiology, Amsterdam, the Netherlands; b Department of Medicine, Division of Cardiology, University of Washington; and King County Emergency Medical Services, Seattle, WA, USA; c Sunnybrook Centre for Prehospital Medicine, Sunnybrook Research Institute and Department of Emergency Services, Sunnybrook Health Science Centre, Division of Emergency Medicine, Department of Family and Community Medicine, Temerty Faculty of Medicine, University of Toronto, Toronto, Ontario, Canada; d Department of Pathophysiology and Transplantation, University of Milan, Italy, Fondazione IRCCS Ca’ Granda Ospedale Maggiore Policlinico, Milan, Italy; e Department of Medicine, Division of Cardiovascular Diseases, University of Alabama at Birmingham, Birmingham, AL, USA

## Abstract

**Introduction:**

Effective defibrillation lies at the heart of successful resuscitation of ventricular fibrillation cardiac arrest. Can it be done better?

**Methods:**

The 50th Anniversary Wolf Creek XVIII Conference was hosted by the Max Harry Weil Institute for Critical Care Research and Innovation in Ann Arbor, Michigan, USA on June 19–21, 2025. Since its inception in 1975, the Wolf Creek Conference has a well-established tradition of providing a unique forum for robust intellectual exchange between thought leaders and scientists from academia and industry focused on advancing the science and practice of cardiac arrest resuscitation.

**Results:**

Innovations in Defibrillation Science was one of six focused panel topics that was presented and discussed by invited panelist and conference participants as recognized thought leaders in the field of cardiac arrest resuscitation, all of whom completed conflict of interest disclosures.

The presentations by invited panelist and discussion focused on four distinct defibrillation-related topics, each written as was presented by its contributing author, providing their individual perspectives. Where applicable, each discussion addressed the current state, potential future state, knowledge gaps, barriers to translation, and research priorities in defibrillation science. Topics included refining the definition of defibrillation and resuscitation success, describing defibrillation mechanisms, double sequential external defibrillation for refractory ventricular fibrillation, and use of quantitative waveform analysis to better direct resuscitation care.

**Conclusions:**

Although much is known, much remains to be learned about defibrillation and its optimal application during resuscitation of cardiac arrest.

## Introduction

This summary of Wolf Creek XVIII’s presentations on defibrillation science focused on distinct topics in the field, each written as presented and discussed at the conference by its contributing author and the audience. It provides a variety of individual perspectives on discrete areas of development, application, and controversy in this exciting field. The discussed topics included: Defining Successful Defibrillation (presented by Rudolph W. Koster, MD, PhD), Basic Mechanisms of Defibrillation (Gregory P. Walcott, MD), Double sequential external defibrillation (Sheldon Cheskes, MD), Quantitative Waveform-Directed Resuscitation (Giuseppe Ristagno, MD, PhD), and Concluding Perspectives (Peter J. Kudenchuk, MD).

## Defining successful defibrillation (Rudolph W. Koster, MD, PhD)

The success of a defibrillation shock was, and still is an important parameter in the quality assessment of modern defibrillators, their waveform, required defibrillation energy, along with the high incidence and importance of recurrent VF after an earlier successful shock.^1^, ^2^, ^3^

A successful defibrillation shock is not the same as a successful resuscitation. For a successful resuscitation, in order of timing and the ultimate success, at least return of an organized rhythm (ROOR) is required, followed by return of spontaneous circulation (ROSC), return of consciousness and return of (near) normal cerebral function. From an electrical perspective, at least removal of VF should be expected after a successful defibrillation shock, and to a certain measure also ROOR.^4^ For ROSC and later parameters of resuscitation success, other factors such as the duration and extend of global myocardial ischemia, the presumed cause of the cardiac arrest and further advanced measures in the pre-hospital and intra-hospital phase are decisive for a good outcome. Therefore, endpoints as ROSC and survival do not serve to assess the quality of the defibrillation process that should include the defibrillator, the electrodes and the recognition and response to unsuccessful or recurrent VF.

If a defibrillation shock fails to convert VF the most likely cause is insufficient current passing through the myocardium to capture a sufficiently large portion of myocardium. The simple solutions in such instances are to increase the shock energy, optimize electrode-to-skin contact and/or their positioning. Reducing myocardial ischemia by extending or shortening the period of CPR before a next shock can also be an option and quantitative waveform analysis (QWA) may play a role to determine an optimal moment for the next shock. If defibrillation fails after repeated shocks it has “pragmatically” been called refractory VF and a new technique Dual Sequential Electrical Defibrillation (DSED) may be considered, based on a single RCT.^5^ A challenge with this “pragmatic” definition of refractory shock is not knowing whether VF was actually successfully terminated but recurred, or remained incessant in the intervening period following shock to the time of the subsequent rhythm assessment (usually 2 min later).

The Guideline algorithms for defibrillation are complex and have changed with the publication of the 2005 Guidelines.^6^ Prior to 2005 each defibrillation shock was followed by a rhythm check and a pulse check. In case the shock was not successful a second and if needed a third shock was given before chest compressions were resumed.^7^ Checking the success of each defibrillation shock came with a price: checking the rhythm and pulse caused a delay of 20–40 s until resumption of chest compressions.^8^ This delay was considered harmful for the probability of survival. Also, it was shown that the probability of a successful shock exceeded 85–90%, while the probability of return of spontaneous circulation (ROSC) immediately after a shock was a few percent only. For this reason, the Guidelines 2005 replaced the three-shock scenario with a single shock scenario, where each shock was immediately followed by resumption of chest compressions, without rhythm- or pulse check. A next rhythm check was performed only after a 2-minutes cycle of cardio-pulmonary resuscitation (CPR). The price for this change in algorithm -not immediately recognized- was, that the success of the shock could only be confirmed after 2 min of CPR and, if VF was encountered 2 min after a previous shock, there remains uncertainty if the shock had been unsuccessful or that there was recurrence of VF. It is possible to distinguish failed defibrillation from recurrent VF with ongoing resuscitation efforts. The immediate defibrillation success at 5 s after the shock can be assessed by inspecting a running paper recording from the defibrillator of the period before shock until >7–10 s after the shock or by filtering techniques that allow on-screen rhythm inspection with ongoing chest compressions. The sensitivity and specificity of these visual inspections during CPR has not been determined.

Because it is not simple during resuscitation to distinguish refractory VF from recurrent VF, a new term emerged: “pragmatic refractory VF”. This term is applied when more than three shocks are needed in sequence during three 2 min cycles of CPR, but unknown if they are for three failed shocks or for recurrent VF. In fact, studies have determined that from all cases with pragmatic refractory VF, in fact only 5–17% had true refractory VF.^9^, ^10^

A summary of generally accepted definitions of outcome after defibrillation is shown in Table 1. There is need for an Utstein-style consensus definition, that is not yet available.

**Table 1:** Generally accepted definitions of defibrillation. outcome.

	**Definition**	**Determinants**	**Alternative definitions**
Successful defibrillation	Absence of VF at 5 s after shock	– Defibrillation energy or voltage gradient in the myocardium – Electrode integrity and position Waveform	– ROOR at 5 s after shock – ROSC at 5 s after shock
Unsuccessful defibrillation	VF at 5 s after shock	Above	Asystole at 5 s after shock
Pragmatic definition of unsuccessful defibrillation	VF at 2 min after shock with uncertainty of a successful prior shock		
Recurrent VF	VF at 2 min after shock after successful shock at 5 s	Above + chest compressions and/or severe myocardial ischemia	−
Refractory VF	Unsuccessful defibrillation at 5 s after shock × 3	Same as unsuccessful defibrillation	−
Pragmatic definition of refractory VF	VF at 4th rhythm analysis after 3 preceding shocks with uncertain success		−
Refractory cardiac arrest	Failure to achieve ROSC after (a) 3 shocks or (b) after a specified duration of ALS	Persistent extensive myocardial ischemia; large ischemic area; occlusion of major coronary artery or main stem; electrolyte disturbances, all resulting in either incessant or continuously recurring VF following shock	−

VF denotes ventricular fibrillation; ROOR return of organized rhythm; ROSC return of spontaneous rhythm; ALS advanced life support; and s seconds.

## Basic mechanisms of defibrillation (Gregory P. Walcott, MD)

### Current state

We have known since 1775 that a large electric shock can manipulate the heart when a Dutch veterinarian used electricity to stop and revive the heart of a chicken.^11^ Defibrillation was first demonstrated in mammals in 1899 by two physiologists.^12^ They showed that small electrical shocks could induce ventricular fibrillation in dogs, and that larger charges would reverse the condition. Since these early experiments, there have been multiple innovations in the field, including moving from AC^13^ to DC^14^current pulses, applying the shock to the body surface rather than directly to the heart, changing the shape of the defibrillation pulse from monophasic to biphasic,^15^, ^16^, ^17^ reducing the size of the defibrillator, and making it simpler to operate so that it can be used by the lay public.^18^

**Fig. 1:**
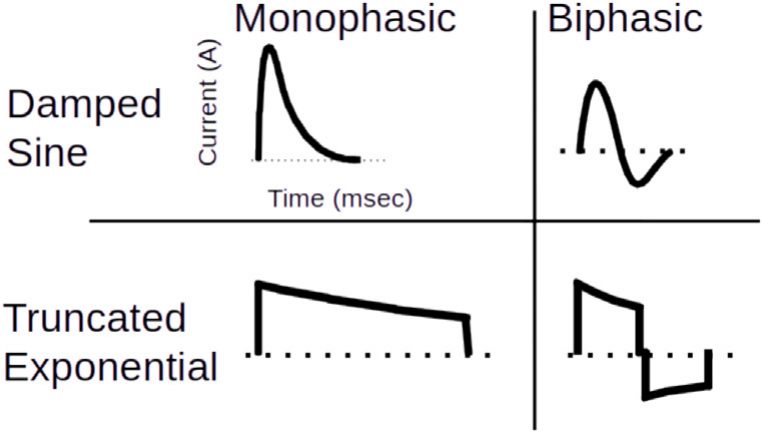
**Types of defibrillator waveforms**. Examples of different defibrillation waveforms. For monophasic waveforms, the current always flows from one electrode (anode) to a second electrode (cathode). For biphasic waveforms the anodal and cathodal electrodes swap partway through the waveform delivery. Damped sine waveforms are rounded using an inductor in the waveform circuit. Truncated exponential waveforms are also referred to as capacitor discharge waveforms. Damped sine and truncated exponential waveforms have similar efficacy. Contemporary defibrillators use capacitor discharge waveforms for technical reasons.

There are some practical facts about defibrillation that make it easier to understand. Defibrillation can be separated into two parts. The first aspect of defibrillation is the shock waveform. Modern defibrillators mostly use some variation of biphasic truncated exponential waveforms (Fig. 1). For a biphasic shock, the electrical current flows in two directions through the heart, with the current's polarity reversing partway through. The two phases of the shocks perform different tasks for defibrillation. The first phase of the shock stops the fibrillation. The second phase keeps the fibrillation from restarting. The characteristics of an ideal first phase for a biphasic waveform can be simulated using models of electrical stimulation of excitable tissue.^19^, ^20^ Defibrillation follows a strength-duration relationship similar to electrical stimulation. The models show that waveform current rather than energy or voltage is the most useful measure of a defibrillation shock. Many external defibrillators adjust the duration of phase 1 of a biphasic shock using the transthoracic impedance measured through the electrodes. As impedance increases, the optimal phase 1 duration also increases, but beyond a point does not lead to increased shock efficacy.^19^ The characteristics of an ideal second phase of a biphasic shock removes enough charge from the myocyte cell membrane to return the transmembrane potential to zero.

The second aspect of defibrillation is the shock distribution through the heart. Electric field (V/cm, potential gradient) is the term used to describe the shock distribution through the heart. It is a surrogate for tissue current density which cannot easily be directly measured. Shock fields are relatively homogeneous for transthoracic shocks with the ratio of lowest to highest potential gradient being 20:1.^21^ For transthoracic shocks, only 4–10% of shock current ever reaches the heart.^22^ A majority of the current dissipates between the two electrodes through the chest wall. Studies have shown that the minimum electric field during a shock needs to be above a specific value for the shock to successfully defibrillate the myocardium. For monophasic waveforms, that value is ∼6 V/cm. For biphasic waveforms, that value is ∼4 V/cm.^23^ Changing the position of the shock electrodes changes the location of the minimum potential gradient.^21^

**Fig. 2:**
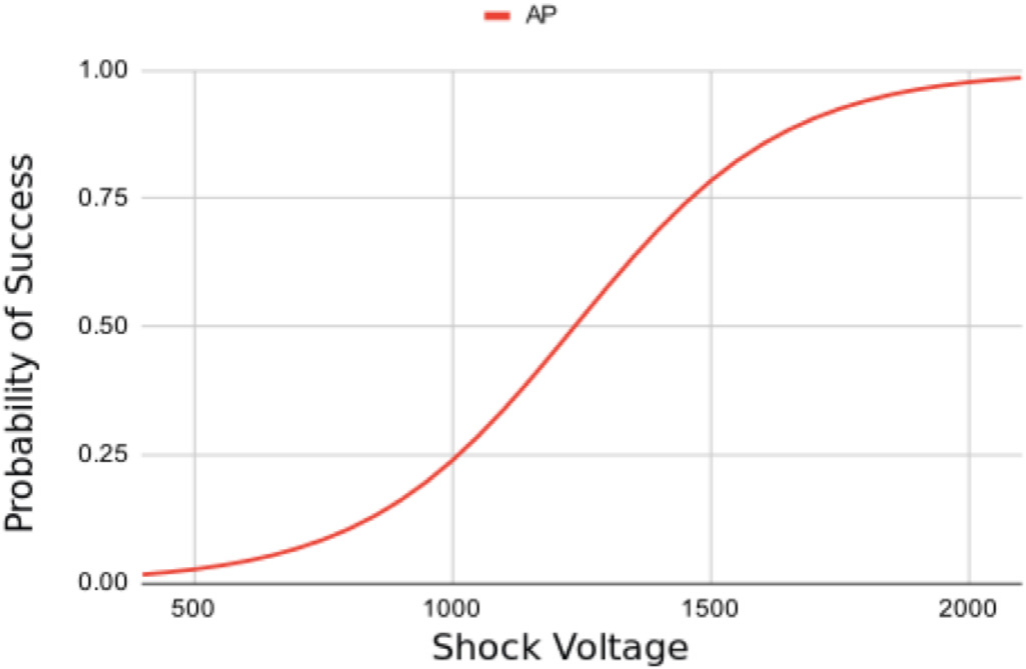
**Hypothetical probability of success curve for defibrillation**. X-axis is transthoracic shock voltage. Y-axis is the probability of a shock being successful (0 – never successful, 1 – nearly always successful).

Much like drug dosing, defibrillation follows a sigmoidal dose response probability of success relationship; smaller shocks have a lower probability of success while larger shocks have a higher probability of success (Fig. 2).^24^, ^25^

Shock failure is a well-known problem for defibrillation, whether using internal or transthoracic shocks, or whether it is for atrial fibrillation or ventricular fibrillation.^26^ Some patients have high defibrillation thresholds (shock level necessary to defibrillate) whether because of body habitus (electrode position with respect to anatomy), because of underlying heart pathology, drugs, or both.^27^ The idea of delivering two shocks across different sets of electrodes (dual defibrillation) instead of delivering with a single pair has been explored.^28^, ^29^, ^30^ One hypothesis is that the second shock raises the shock field strength in the area of the heart that is low for the first shock.^31^ For transthoracic defibrillation in a recent swine mapping study, delivering a shock through the right-left lateral electrodes places the minimum potential gradient in the posterior basal area of the left ventricle.^21^ Delivering a shock from anterior-posterior electrodes places the minimum potential gradient in the left and right lateral portions of the heart. In a separate study, delivering a shock through the right-left lateral electrodes followed by a shock through the anterior-posterior electrodes defibrillated with a higher success rate than shocking through either electrode pair alone.^29^

### Potential future state

The ideal future state would be personalized defibrillation where a defibrillator could predict what shock size would be necessary to defibrillate a patient. The fibrillating heart uses three times as much energy as the non-fibrillating heart.^32^ Present-day defibrillators used under current resuscitation guidelines terminate VF in 90–95% of patients with 3 or fewer shocks.^9^ A challenge is what to do with the remaining patients. Studies have shown that defibrillators delivering larger shocks have higher defibrillation success rates compared to smaller shocks.^33^, ^34^

An important point to remember though is that anything done to improve the result of the 5–10% of pragmatic refractory ventricular fibrillation patients must not result in fewer patients in the 90–95% of patients that are being successfully defibrillated. Some combination of ECG analysis and electrode impedance assessment to predict myocardial affinity to defibrillation and shock field characteristics may allow for more focused defibrillation methodology in patients.

### Knowledge gaps

Several questions need to be answered in order to improve defibrillation efficacy and resuscitation outcomes. Biphasic waveform shapes have been optimized based on population studies. Would specific patients benefit from shorter or longer duration pulses? How do we best increase the shock field strength throughout the heart? A concern with this strategy is that the larger shock will cause damage to the heart and keep it from returning to function. Optimizing the shock electrode position may do this as could delivering two shocks through different electrode pairs. What is the best strategy remains to be determined.

### Barriers to translation

A major challenge for studying the question of high defibrillation thresholds and the occurrence of pragmatic refractory ventricular fibrillation is the lack of a model to study. Both computer and pre-clinical models have a homogeneity that makes studying outliers difficult. The lack of a mechanism(s) explaining why some patients are difficult to defibrillate and/or are difficult to maintain in a non-shockable rhythm makes this problem difficult to solve.

### Priorities for research and implementation

Defibrillators have improved significantly over the last century both technically as well as physiologically. The great majority of patients are successfully defibrillated. Future improvements will likely involve how to treat patients for whom this is not the case. Additionally, better integration of defibrillation with the rest of the resuscitation protocol may lead to better resuscitation outcomes.

## Double sequential external defibrillation (Sheldon Cheskes, MD)

**Fig. 3:**
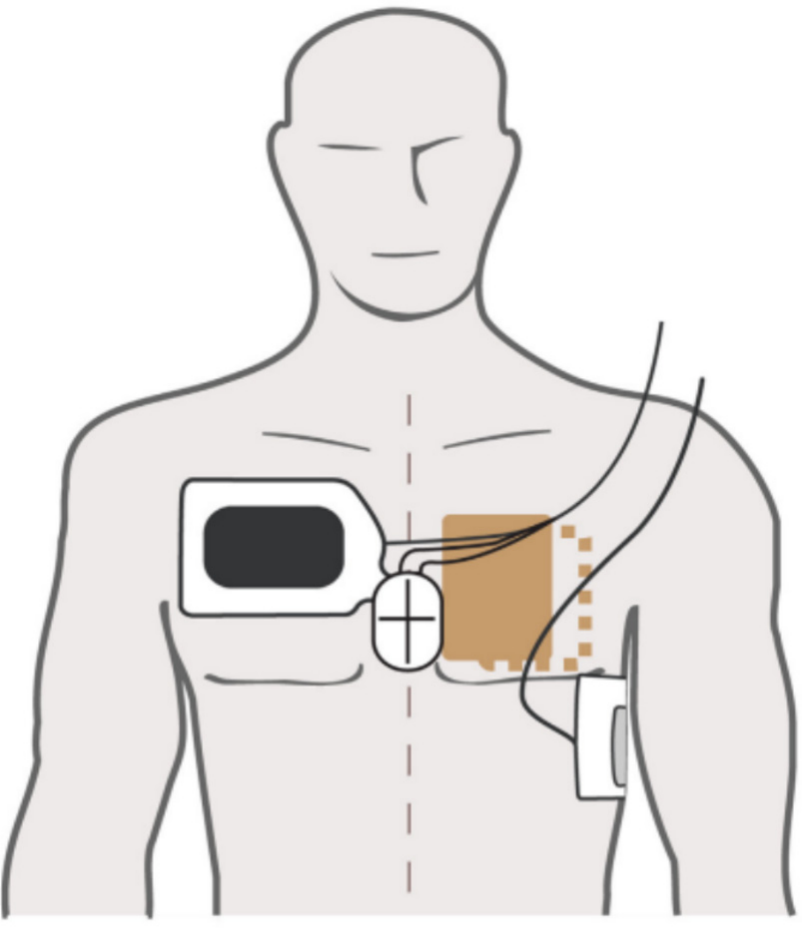
**Pad position for double sequential external defibrillation**. Anterior (black)–Lateral (gray) represents anterior-lateral pad position. Anterior (orange)–Posterior (dotted orange) represents anterior-posterior pad position. (For interpretation of the references to colour in this figure legend, the reader is referred to the web version of this article.)

Double sequential external defibrillation (DSED) is a resuscitation technique involving the rapid delivery of two shocks from two defibrillators, with pads placed in anterior-lateral (AL) and anterior-posterior (AP) positions (Fig. 3). This approach has gained a great deal of notoriety following the publication of the Double Sequential External Defibrillation for Refractory Ventricular Fibrillation (DOSE-VF trial), a cluster-randomized crossover study conducted across six paramedic services in Ontario, Canada. The trial demonstrated that DSED significantly improved outcomes—including VF termination, return of spontaneous circulation (ROSC), survival to hospital discharge, and neurologically intact survival—compared to standard defibrillation.^5^ These findings informed the 2023 ILCOR guidelines, which suggest DSED may be employed as a strategy for refractory VF.^35^

Mechanistically, DSED’s efficacy may be attributed to several factors. One hypothesis is that multiple shock vectors expose a greater proportion of myocardial tissue to defibrillatory current, particularly along the longitudinal axis of cardiac myocytes.^36^ Another explanation involves reduced transthoracic impedance, which increases current density delivered to the myocardium.^37^ Secondary analysis of data from DOSE-VF study revealed a consistent 15–20% lower impedance when using anterior-posterior pads as compared to AL pads in the same patient employing VC shocks and during the same shock for DSED shocks, suggesting enhanced current delivery regardless of defibrillator manufacturer (Table 2).^38^ From a safety perspective sequential shocks, as used in DOSE-VF trial, appear safer for defibrillator integrity than simultaneous shocks, which have been associated with rare documented case of device damage where pad position and indication for DSED differed from that used in the trial.^29^, ^39^, ^40^ Importantly, DSED also appears to shorten the duration of VF—a factor strongly correlated with improved neurological outcomes.^3^, ^41^ DSED has as well been shown to be more effective than standard defibrillation for shock-refractory VF while yielding clinically relevant but non statistically significant improvements for those in recurrent VF.^10^

**Table 2:** Shock energy, impedance and current delivery during the DOSE-VF randomized controlled trial.*

**Shock number**	**Energy (joules) AL pad**	**Impedance (ohms)** **AL pad**	**Current (amps)** **AL pad**	**Energy (joules)** **AP pad**	**Impedance (ohms)** **AP pad**	**Current (amps)** **AP pad**
Shock 1	159.1	94.4	12.9			
Shock 2	202.4	93.5	14.5			
Shock 3	257.0	93.5	16.3			
Shock 4	269.3	93.2	16.6	257.7	70.5	19.0
Shock 5	271.7	92.0	16.9	260.4	70.3	19.1
Shock 6	272.6	92.8	16.9	264.0	69.8	19.3

Where AL = Anterior-Lateral; AP = Anterior-Posterior.

* Calculations for Current based on formulas provided by and calculations performed by both Zoll Medical Inc. and Stryker Emergency Care. AP pad calculations include energy, impedance and current from AP pad during both vector change and double sequential external defibrillation. The calculations of current from Stryker devices (LIFEPAK15) only include current delivery during first 6 ms of pulse duration.^38^

### Potential future state

The future of DSED lies in its potential to be applied earlier in the resuscitation sequence. Several ongoing randomized controlled trials—including DOUBLE-D (ClinicalTrials.gov ID NCT06447805), STRAT-DEFI (ClinicalTrials.gov ID NCT06781892), DUALDEFIB (ClinicalTrials.gov ID NCT06672159), and DOSE-VF2—are investigating the impact of DSED when delivered after a single failed shock or even as the initial defibrillation strategy. These studies may redefine its role, shifting DSED from a rescue therapy to a frontline intervention. Technological innovation will play a key role in facilitating broader adoption. Future defibrillators may incorporate four-pad systems with automated sequencing, reducing the need for manual coordination and minimizing the risk of device damage. Integration with mechanical CPR and AEDs could further expand DSED’s accessibility, making it feasible for a wider range of EMS systems, including those with limited resources.^42^ Artificial intelligence tools are also on the horizon. Machine learning algorithms capable of analyzing ECG data in real time may soon predict refractory VF, allowing for tailored defibrillation strategies. Coult et al. demonstrated that predictive models could identify patients at high risk for shock-refractory VF, potentially enabling earlier application of DSED.^43^ Similarly, amplitude spectrum area (AMSA) analysis may guide shock timing and energy delivery, offering a more personalized approach to resuscitation.^44^.

### Knowledge gaps

Despite the promising results of DOSE-VF, several critical questions remain unanswered. The optimal timing and interval between DSED shocks are still under investigation. Preliminary data suggest that shorter intervals may be more effective, but the balance between efficacy and device safety must be carefully considered.^29^, ^45^ The precise mechanisms underlying DSED’s benefit—whether due to vector exposure, impedance reduction, or current density—requires further validation. While impedance data from DOSE-VF suggest greater current delivery occurs through anterior-posterior pads, the relationship between current and clinical outcomes remains to be fully elucidated. Additionally, the comparative efficacy of DSED versus vector change (VC) defibrillation is unclear.^46^ The influence of pad positioning and defibrillator waveform characteristics on outcomes also warrants further exploration. Recent ILCOR consensus statements have called for more research into pad size and placement, particularly in pediatric and obese populations.^47^ Long-term neurological outcomes and cost-effectiveness analyses of DSED are also currently limited.

### Barriers to translation

Several barriers hinder the widespread implementation of DSED. Chief among them are concerns about defibrillator damage, particularly with simultaneous shocks. Manufacturer warranty exclusions have contributed to hesitancy among EMS providers.^48^ However, data from DOSE-VF and subsequent surveys indicate that damage is exceedingly rare and primarily associated with simultaneous shocks, not the sequential technique used in the trial.^39^ Logistical challenges also exist. Many EMS systems may not routinely carry two defibrillators on scene, especially in rural or resource-constrained settings. Training gaps among providers—including proper pad placement, shock sequencing, and integration with mechanical CPR—further complicate implementation. Regulatory hurdles (DSED is currently an off label use of a defibrillator), protocol updates, and equipment standardization add additional layers of complexity. Moreover, the perception that DSED requires two manual defibrillators has been challenged by evidence from the DOSE-VF trial, which successfully employed combinations of manual and AED devices.^49^

### Priorities for research and implementation

To advance the field, several research and implementation priorities must be addressed. Confirmatory randomized trials are needed to compare early DSED to standard and VC defibrillation strategies. These studies should explore not only survival outcomes but also neurological recovery and quality of life. Investigations into pad positioning,^10^ shock timing, and waveform characteristics will help optimize clinical outcomes. Standardized training modules for EMS providers are essential. These could include potential integration with AEDs and mechanical CPR, as well as guidance on device combinations and pad placement. Engagement with manufacturers to design defibrillators optimized for DSED and to clarify warranty policies based on emerging evidence will be critical. Finally, understanding the economic and logistical implications of DSED will help guide policy decisions and ensure that this promising intervention reaches the patients who need it most.

## Quantitative waveform-directed resuscitation (Giuseppe Ristagno, MD, PhD)

### Current state

Cardiopulmonary resuscitation (CPR) guidelines recommend a fixed, time-based defibrillation strategy in which rhythm analysis and, if indicated, shock delivery occur every two minutes, regardless of myocardial status.^50^ This approach requires cyclical interruptions of chest compressions (CCs), which reduces myocardial and cerebral perfusion, lowering the likelihood of successful defibrillation and worsening outcomes.^51^, ^52^, ^53^, ^54^, ^55^

**Fig. 4:**
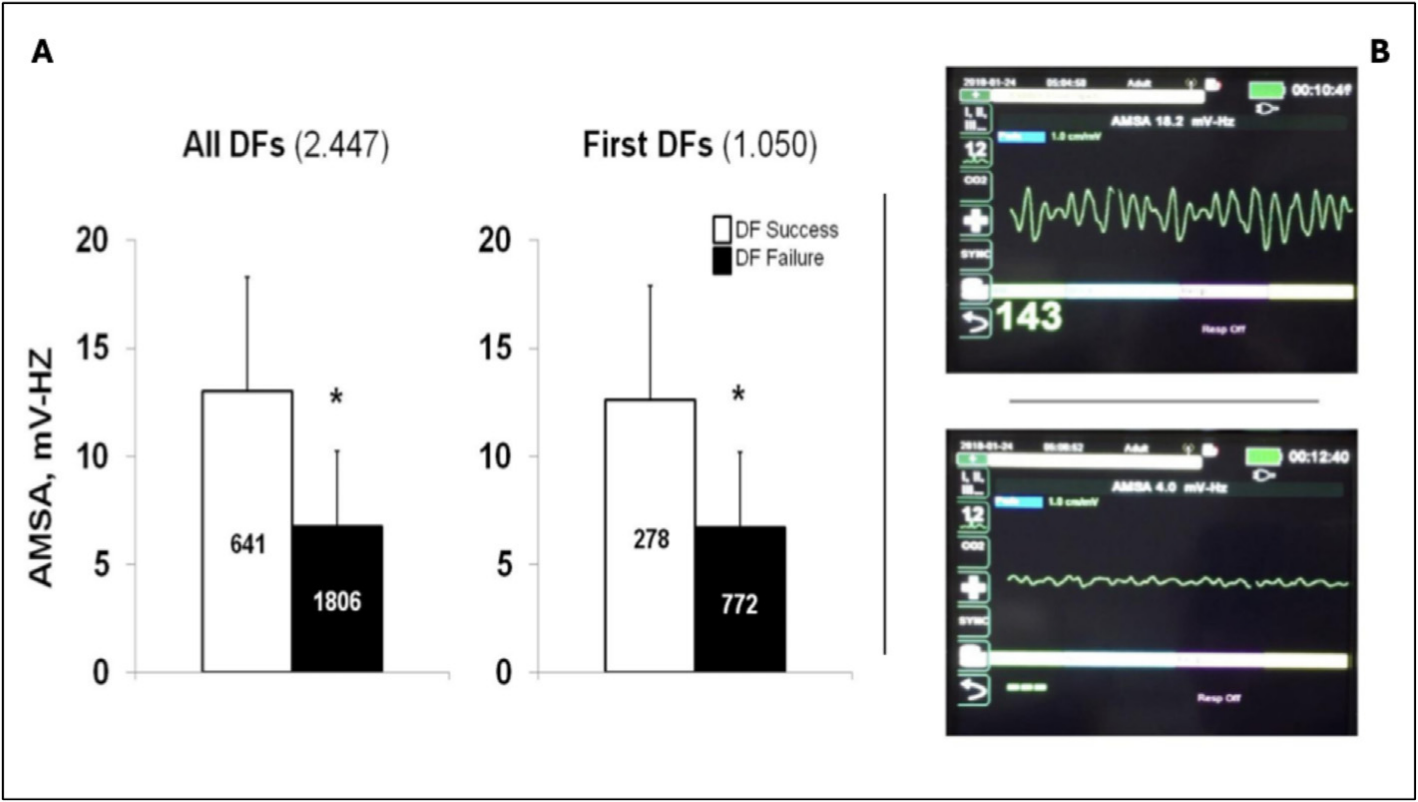
**AMSA prediction of defibrillation success**. (A) AMSA in successful and unsuccessful defibrillations (DF) for all and first attempts. Numbers in brackets represent the number of defibrillations attempts.^58^ *P < 0.0001 between successful and unsuccessful defibrillations. (B) Examples of display showing real-time AMSA values (from high, on the top, to low AMSA on the bottom) from the experimental defibrillators (modified X-Series ZOLL Med. Corp., USA) used in the AMSA trial.^44^

Real-time ventricular fibrillation (VF) waveform analysis has been proposed as a non-invasive method to guide defibrillation during CPR and avoid ineffective shocks.^54^ Numerous algorithms have been developed to optimize defibrillation timing, including: time domain features (root mean square amplitude, mean and median slope, Vrhythm, signal integral); frequency domain parameters (peak and median frequency, spectral flatness measure, amplitude spectrum area (AMSA), spectral energy, power spectrum analysis); nonlinear dynamic measures (detrended fluctuation analysis, angular velocity, entropy, Hurst exponent); and other algorithms combining two of more of the previous methods with machine learning, neural networks, or support vector machine.^56^, ^57^ Among these, AMSA has emerged as an accurate predictor of defibrillation outcome in out-of-hospital cardiac arrest (OHCA)^58^, ^59^, ^60^ (Fig. 4A), with proposed thresholds of ≥15.5 mV-Hz for success and <6.5 mV-Hz for failure.^58^

Despite extensive retrospective research, only two waveform-based algorithms have been prospectively tested.^44^, ^61^ A randomized controlled trial (RCT) comparing a standard AED protocol with a Vrhythm-guided shock protocol in 987 VF-OHCA patients assigned each a numeric VF score and recommended the delivery of an immediate shock or a 2-min CPR if the score exceed a predefined threshold or not, respectively. Delaying defibrillation guided by the waveform score did not result in better outcome for survival to discharge, ROSC or survival to admission. It was noted that patients whose VF score increased during CPR showed significantly better short-term outcomes, compared to those whose score did not improve.^61^ More recently, a small multicenter RCT evaluated AMSA-guided CPR was terminated early and remained underpowered for relevant endpoints. Not surprising, no significant benefit from AMSA-guided defibrillation was demonstrated for successful defibrillation but demonstrated the feasibility of real-time AMSA determination.^44^ Indeed, AMSA was calculated and its value displayed in the monitor defibrillator during pauses for ventilation. Fig. 4B reports two real cases of VF, coarse and fine, with corresponding AMSA values. AMSA ≥15.5 mV-Hz predicted defibrillation success (positive predictive value: 0.77), while <6.5 mV-Hz predicted failure (negative predictive value: 0.86). Higher AMSA values were also associated with favorable long-term neurological outcomes.^62^

### Potential future state

The future application of ECG waveform analysis has the potential to transform CPR into a dynamic and personalized intervention, moving beyond rigid, time-based protocols (Table 3). Future algorithms could tailor treatment, based on the predictive score measured real-time, minimizing CC interruptions and reducing futile, potentially harmful shocks.^44^, ^50^, ^58^, ^62^, ^63^, ^64^

**Table 3:** Potential uses and knowledge gaps of ECG waveform rhythm analysis during cardiopulmonary resuscitation.

**Potential uses**	**Knowledge gaps**
VF waveform-guided defibrillation	Need for large-scale prospective studies
Optimization of shock energy	Optimization of decision thresholds
Continuous CPR quality monitoring	Limitations of current real-time measurement
Prediction of STEMI	Impact of pads placement
Targeted management of refractory VF	Personalized approaches

STEMI, ST-elevation myocardial infarction; VF, ventricular fibrillation.

Waveform analysis may also guide selection of optimal defibrillation energy in the future. High AMSA values were shown to predict successful low-energy shocks (150 J), whereas lower AMSA required higher energy (300–360 J).^64^ VF analysis could be also used to provide real-time feedback on CC quality to rescuers. Both animal models and clinical data showed that AMSA decreases between shocks during CPR with inadequate compression depth (i.e. <5 cm), while increases with deeper, high-quality CC.^44^, ^54^, ^59^, ^60^, ^62^, ^63^, ^64^

Dynamic VF waveform changes might offer diagnostic insights into the underlying cause of arrest,^44^ as suggested by a retrospective study of 754 OHCA cases where AMSA values were significantly lower in patients with STEMI.^65^ These findings support the concept of future “diagnostic defibrillators” that could not only guide resuscitation but also assist in early identification of conditions requiring specific interventions, such as percutaneous coronary intervention.^65^, ^66^, ^67^

Potentially, ECG waveform analysis could distinguish between refractory VF or “recurrent VF”,^5^, ^68^, ^69^ as shown by a recent study of 360 cases, in which AMSA values were significantly lower in true shock-refractory VF compared with recurrent VF. These findings support the use of VF analysis as additional decisional support to tailor potential rescue strategies.^5^, ^50^, ^69^ Finally, combining waveform analysis with artificial intelligence (AI) and deep neural networks may enable continuous rhythm assessment during CCs, achieving >90% sensitivity and enabling real-time, AI-driven CPR guidance.^70^, ^71^, ^72^

### Knowledge gaps

Several knowledge gaps must be addressed (Table 3). The first pertains to prospective evidence. Prospective studies so far failed to demonstrate clinically relevant benefit. Second, the orientation of ECG recording has been shown to significantly influence AMSA values.^71^ Thus, different pad placement might significantly affect accuracy of the proposed AMSA threshold,^73^, ^74^ underscoring the need for standardized or compensatory approaches. Finally, patient-specific factors may influence waveform analysis algorithms’ performance. Retrospective studies indicate that comorbidities, cardiovascular medications, and STEMI, lead to variable AMSA values and influence its variance during CPR.^44^, ^75^, ^65^, ^66^, ^67^

### Barriers to translation

The first challenge is achieving accurate, real-time ECG rhythm analysis during ongoing CC without motion artifacts.^71^, ^72^, ^73^ Although adaptive filters and algorithms show promise, further technological refinement and validation in real-world settings are essential.^71^, ^72^

Technical variability further complicates implementation. Differences in defibrillator manufacturers, sampling rates, and filter settings across prehospital systems, together with patient-specific factors can hinder multicenter adoption and the development of universally applicable algorithms.^44^, ^56^, ^60^, ^65^, ^66^, ^67^

Deploying “smart defibrillators” also requires substantial investment, regulatory approval, and comprehensive EMS training. In the AMSA trial, for instance, EMS providers underwent structured training and re-training including didactic sessions and hands-on workshops on pad placement, hand positioning to minimize artifacts, AMSA interpretation and recognition of thresholds, and deriving interventions.^44^

### Priorities for research and implementation

The priority is conducting adequately powered, multicenter RCTs to determine whether ECG waveform–guided CPR improves survival with good neurological outcomes compared with current protocols.^44^, ^61^ A second priority is developing and validating advanced signal-processing and AI–based algorithms capable of continuous ECG analysis during ongoing CC without pauses for artifact suppression.^71^, ^72^

Research should also examine VF score thresholds across diverse clinical and patient characteristics, supporting individualized defibrillation guidance and the evolution of “smart diagnostic defibrillators” able to optimize timing, energy, and etiology recognition and suggest intervention priorities or management pathways.^58^, ^75^

Finally, combining waveform analysis with other physiological indicators, such as end-tidal CO_2_ could enhance assessment of myocardial and systemic perfusion during CPR, promoting personalized resuscitation and improved post-arrest care (Table 4).^76^

**Table 4:** Barriers to implementation of ECG waveform rhythm analysis during cardiopulmonary resuscitation and priorities for research and implementation.

**Barriers to translation**	**Priorities for research and implementation**
Technological limitations in CC-induced artifact removal	Large-Scale Randomized Controlled Trials
Integration into existing guidelines and clinical protocols	Validate Continuous ECG Analysis Algorithms
Cost and availability of specialized equipment	Refine and Standardize VF Waveform Algorithms (i.e. AMSA) Thresholds
Training and education for rescuers	Integrate VF waveform algorithms with Multimodal Physiological Monitoring
Challenges with data heterogeneity and Influence of underlying pathologies	Develop “Diagnostic Defibrillators”

## Concluding perspectives (Peter J. Kudenchuk, MD)

Successful defibrillation involves the complex interplay of patient substrate with operational factors, including meticulous procedural technique, shock energy, waveform, shock vector, shock timing, and probability. Defining defibrillation success can itself be challenging because the resulting cardiac rhythm may be obscured by artifact from immediately-resumed CPR, can evolve over time, and not necessarily be one-in-the-same as that observed when CPR is paused 2 min later for rhythm and pulse assessment.

Numerous studies have demonstrated that the challenge confronting defibrillation is not shock’s immediate success in terminating VF, but rather VF’s recurrence following such success.^1^, ^77^, ^78^ Such recurrence is responsible for 80–95% of post-shock VF that incrementally returns (unseen) during the ensuing 2 min period of CPR after a successful shock.^9^ These observations question the applicability of double sequential defibrillation (DSED) for the majority of patients with so-called shock-refractory (but actually recurrent) VF, since in this latter group DSED has not demonstrated a statistically significant survival benefit.^10^ DSED’s effectiveness requires confirmation, including whether its benefit, if any, is strictly confined to the relatively small minority of patients with truly incessant (never terminating) VF.

A recent publication stemming from a “thinktank” co-convened by the American Heart Association, the Cardiac Safety Research Consortium and the Food and Drug Administration addressed DSED, the potential benefits of this technology, along with limitations in the design of current studies and their analysis.^79^ Ongoing clinical trials (not to mention current clinical practice) need to insure that well-intentioned efforts to improve resuscitation outcome by wider or earlier application of DSED do not adversely influence the prognosis of those already being successfully defibrillated and who may instead benefit from alternate approaches to address and prevent VF’s recurrence. To this end, the 2025 AHA Guidelines state that the usefulness of double sequential defibrillation for adults in cardiac arrest with persisting VF/pulseless VT after ≥3 consecutive shocks has not been established and requires further investigation.^80^.

So, how do we become “smarter” about optimizing defibrillation, an already highly effective therapy? VF waveform analysis offers an exciting future prospect. This uses a variety of technical approaches, ideally performed during uninterrupted CPR, to identify the underlying cardiac rhythm and guide resuscitation efforts in real time, with the potential to predict the response of VF to shock, suggest the optimal timing for defibrillation, and direct use of ancillary therapies to maximize their success and prevent recurrences.^43^ This new chapter in resuscitation, guided by real-time physiology, may well pave the future path in what, how, when, and in whom, we can do everything better.

## CRediT authorship contribution statement

**Rudolph W. Koster:** Writing – review & editing, Writing – original draft, Conceptualization. **Peter J. Kudenchuk:** Writing – review & editing, Writing – original draft, Conceptualization. **Sheldon Cheskes:** Writing – original draft. **Giuseppe Ristagno:** Writing – original draft. **Gregory P. Walcott:** Writing – original draft.

## Declaration of competing interest

Dr. Cheskes received grant funding from HSF Canada, Zoll Medical and the Cardiovascular Network of Canada (CANET). He reports having received honorarium from Zoll Medical for educational speaking on resuscitation science. Dr. Koster is a paid consultant for Stryker Emergency Care. Dr. Kudenchuk reports funding from National Institutes of Health (NIH) as Principal Investigator for the SIREN Network at the University of Washington. He is member of the Editorial Board for Resuscitation Journal. Dr. Ristagno reports research support from Philips North America and membership of the Editorial Board of Resuscitation Plus. Dr. Walcott reports grant support from Stryker Emergency Care and has joint patents with Stryker Emergency Care.
